# Formate Promotes *Shigella* Intercellular Spread and Virulence Gene Expression

**DOI:** 10.1128/mBio.01777-18

**Published:** 2018-09-25

**Authors:** Benjamin J. Koestler, Carolyn R. Fisher, Shelley M. Payne

**Affiliations:** a Department of Molecular Biosciences and Institute for Cellular and Molecular Biology, The University of Texas at Austin, Austin, Texas, USA; Princeton University

**Keywords:** *Shigella*, formate, metabolism, virulence regulation

## Abstract

The intracellular human pathogen Shigella flexneri invades the colon epithelium, replicates to high cell density within the host cell, and then spreads to adjacent epithelial cells. When S. flexneri gains access to the host cytosol, the bacteria metabolize host cytosolic carbon using glycolysis and mixed acid fermentation, producing formate as a by-product. We show that S. flexneri infection results in the accumulation of formate within the host cell. Loss of pyruvate formate lyase (PFL; Δ*pflB*), which converts pyruvate to acetyl coenzyme A (CoA) and formate, eliminates S. flexneri formate production and reduces the ability of S. flexneri to form plaques in epithelial cell monolayers. This defect in PFL does not decrease the intracellular growth rate of S. flexneri; rather, it affects cell-to-cell spread. The S. flexneri Δ*pflB* mutant plaque defect is complemented by supplying exogenous formate; conversely, deletion of the S. flexneri formate dehydrogenase gene *fdnG* increases host cell formate accumulation and S. flexneri plaque size. Furthermore, exogenous formate increases plaque size of the wild-type (WT) S. flexneri strain and promotes S. flexneri cell-to-cell spread. We also demonstrate that formate increases the expression of S. flexneri virulence genes *icsA* and *ipaJ*. Intracellular S. flexneri *icsA* and *ipaJ* expression is dependent on the presence of formate, and *ipaJ* expression correlates with S. flexneri intracellular density during infection. Finally, consistent with elevated *ipaJ*, we show that formate alters S. flexneri-infected host interferon- and tumor necrosis factor (TNF)-stimulated gene expression. We propose that *Shigella*-derived formate is an intracellular signal that modulates virulence in response to bacterial metabolism.

## Abstract (precis)

*Shigella* is an intracellular pathogen that invades the human host cell cytosol and exploits intracellular nutrients for growth, enabling the bacterium to create its own metabolic niche. For *Shigella* to effectively invade and replicate within the host cytoplasm, it must sense and adapt to changing environmental conditions; however, the mechanisms and signals sensed by S. flexneri are largely unknown. We have found that the secreted *Shigella* metabolism by-product formate regulates *Shigella* intracellular virulence gene expression and its ability to spread among epithelial cells. We propose that *Shigella* senses formate accumulation in the host cytosol as a way to determine intracellular *Shigella* density and regulate secreted virulence factors accordingly, enabling spatiotemporal regulation of effectors important for dampening the host immune response.

## INTRODUCTION

Shigella flexneri is an enteropathogenic subspecies of Escherichia coli that causes shigellosis, an acute mucosal inflammation resulting in severe bloody dysentery. After ingestion, *Shigella* traverses the digestive tract to the colon and crosses the colonic epithelium by exploiting M cells (1); the bacteria then invade the basolateral face of the epithelium using a contact-dependent type 3 secretion system (T3SS) encoded on a virulence plasmid, causing epithelial cells to engulf the bacteria. After *Shigella* enters the cell and escapes the host engulfment vacuole, it multiplies within the host cell cytoplasm and subsequently spreads to adjacent cells using the protein IcsA (also known as VirG), which catalyzes host actin synthesis, propelling the bacterium into neighboring cells (2, 3).

Expression of S. flexneri virulence genes inside the host epithelial cell is dynamic. Although initially required for invasion, T3SS genes are repressed upon entry into the host epithelial cell (4–6). The T3SS genes and additional cell-to-cell spread genes are later reactivated through an unidentified mechanism immediately prior to spread (6). S. flexneri expresses a suite of T3SS effectors to dampen the host response to cytosolic infection. The effectors IpgD, OspI, OspG, OspF, and IpaH work in concert to modulate inflammation (7, 8). Host intracellular trafficking, which alters both epithelial cell homeostasis and defense against cytosolic bacteria, is another target of S. flexneri*-*secreted effectors. The secreted effector IpaB redirects host cholesterol away from the Golgi apparatus, resulting in Golgi fragmentation (9). Likewise, the secreted effectors VirA and IpaJ alter Golgi apparatus-mediated cell trafficking (10, 11). IpaJ is a cysteine protease that alters N-myristoylation (11), ultimately inhibiting the trafficking of STING, a known host cytosolic sensor of S. flexneri infection (12, 13). This results in blocking the STING-mediated activation of the type I interferon response, including cytokines such as CXCL10 (11, 12).

S. flexneri differentially regulates over a quarter of its genes in the intracellular environment compared to S. flexneri grown *in vitro*, including many genes involved in central carbon metabolism (5, 14). S. flexneri tricarboxylic acid (TCA) cycle enzymes are repressed in the intracellular environment, whereas enzymes involved in glycolysis and mixed acid fermentation pathways are increased and necessary for virulence (15–17). Specifically, *pflB*, the gene encoding pyruvate formate lyase (PFL), and PFL-associated genes are upregulated in the host cell (5, 15). PFL converts pyruvate to acetyl coenzyme A (CoA), producing formate as a by-product. An S. flexneri Δ*pflB* mutant is defective in plaque formation; however, deletion of *pflB* does not impact S. flexneri growth rate within the host cell (15, 16).

The scope of this study was to determine the role of S. flexneri formate metabolism in virulence during the host-cytosolic phase of infection. We propose a model where *Shigella*-derived formate accumulates in the host cell cytoplasm and promotes the expression of IcsA and IpaJ, linking S. flexneri metabolism and intracellular cell density to intercellular spread and host-pathogen response.

## RESULTS

### Formate promotes S. flexneri plaque formation in Henle-407 monolayers.

**FIG 1:**
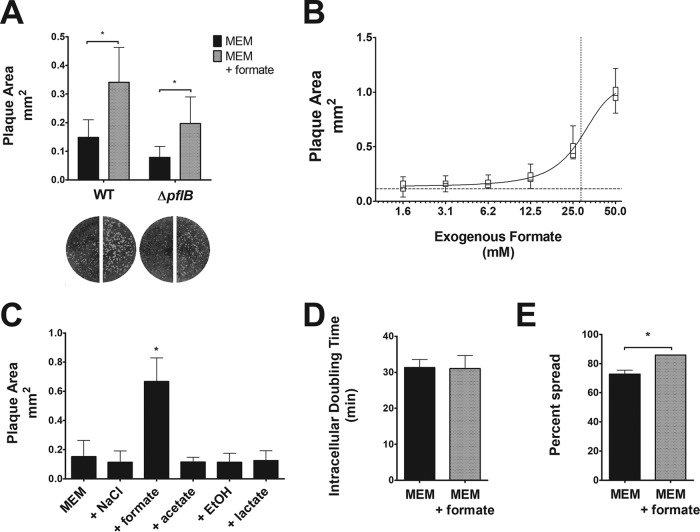
Formate-induced increase in S. flexneri cell-to-cell spread. (A) Wright-Giemsa stain of Henle-407 monolayers infected with S. flexneri and S. flexneri Δ*pflB* mutant with or without 20 mM formate. Plaque sizes were measured; an asterisk indicates statistical significance. (B) Plaque size of S. flexneri was measured when various concentrations of formate were added as supplements. The dotted line on the *y* axis indicates the plaque area when no formate was added as a supplement (0.1 mm^2^), while the dotted line on the *x* axis indicates the 50% effective concentration (28.4 mM). (C) Plaque size of S. flexneri was measured with 20 mM NaCl, formate, acetate, ethanol, or lactate; an asterisk indicates a statistically significant difference from MEM. Only formate was found to increase S. flexneri plaque size. (D) S. flexneri intracellular doubling time was measured in Henle-407 cells with or without 20 mM formate. (E) S. flexneri cell-to-cell spread was measured at 3 hpi with or without 20 mM formate; an asterisk indicates statistical significance.

One measure of S. flexneri virulence is the ability of S. flexneri to form plaques in cultured epithelial cell monolayers, which is contingent on both the ability of S. flexneri to replicate intracellularly and its ability to spread to adjacent host cells (18). Previous studies have shown that failure to form plaques, or formation of smaller-than-wild-type (WT) plaques, correlates with decreased virulence in animal models (18–20). An S. flexneri Δ*pflB* mutant forms smaller plaques in Henle-407 monolayers than does WT S. flexneri (15). Consistent with a previous study (16), the S. flexneri Δ*pflB* mutant showed no intracellular growth defect (see Fig. S1 in the supplemental material). Although not statistically different, the doubling time of the mutant was slightly shorter; as such, any effect of this small difference in growth over the time of plaque formation would be expected to lead to larger, rather than smaller, plaques. This suggested that formate, a product of PFL, secreted by S. flexneri promoted plaque formation independently of bacterial growth. If the plaque defect in the *pflB* mutant was due solely to the loss of formate, providing exogenous formate should rescue the Δ*pflB* plaque defect. At biological pH, formate cannot passively diffuse across bacterial or eukaryotic cell membranes (21). However, a previous study showed that exogenously supplemented formate could be taken up by cultured cells (22), and we confirmed that that Henle-407 cells take up radiolabeled formate when supplemented in tissue culture medium (Fig. S2). We supplemented tissue culture medium of cells infected with the S. flexneri PFL mutant (Δ*pflB*) with 20 mM formate after S. flexneri invasion and measured plaque formation. In the absence of exogenous formate, the average Δ*pflB* plaque size was approximately half the size of the WT strain (Fig. 1A), consistent with previous observations (14). However, with the addition of exogenous formate, the plaque size of the Δ*pflB* mutant was restored to WT size (Fig. 1A), suggesting that S. flexneri*-*derived formate promotes S. flexneri plaque formation. Additionally, the plaque size of the WT strain increased 2.3-fold in the presence of exogenous formate (Fig. 1A). The formate concentration used in these experiments is in the midrange of the dose-response curve (Fig. 1B) and is within the range found in the mammalian gastrointestinal tract (23–26).

10.1128/mBio.01777-18.1 FIG S1 S. flexneri intracellular doubling times of the WT and *ΔpflB* strains were measured in Henle-407 cells. No significant difference in doubling time was observed between the two strains. Download FIG S1, TIF file, 1.65 MB. Copyright © 2018 Koestler et al. 2018 Koestler et al. This content is distributed under the terms of the Creative Commons Attribution 4.0 International license.

10.1128/mBio.01777-18.2 FIG S2 **[**^14^C]formate uptake was quantified in Henle-407 cells over the course of 48 hours. A significant increase in ^14^C cpm was observed over time. *n* = 3. Download FIG S2, TIF file, 0.85 MB. Copyright © 2018 Koestler et al. 2018 Koestler et al. This content is distributed under the terms of the Creative Commons Attribution 4.0 International license.

To determine if other fermentation by-products promote S. flexneri plaque formation, the medium of S. flexneri-infected Henle-407 cells was supplemented with 20 mM formate, acetate, ethanol, or lactate. With the exception of ethanol, these compounds were provided as supplements as sodium salts; thus, we included a control supplemented with 20 mM NaCl. Exclusively in the presence of formate, we observed a 2.7-fold increase in plaque area (Fig. 1C).

Although we cannot completely rule out an effect of formate on Henle-407 cells, experiments to find such an effect have been negative. Examination of the monolayers by light microscopy revealed no changes in morphology or cell density when Henle-407 cells were grown with 20 mM formate, and there was no effect on medium pH. Additionally, formate had no cytotoxic effects on Henle-407 cells, as measured by lactate dehydrogenase release (Fig. S3A), and formate did not alter the growth of Henle-407 cells (Fig. S3B).

10.1128/mBio.01777-18.3 FIG S3 (A) Uninfected Henle-407 cell cytotoxicity in the presence and absence of exogenous formate (20 mM) was determined by quantifying released lactate dehydrogenase (LDH) over 72 hours. We observed no significant difference in LDH release in the presence and absence of formate. (B) Henle-407 cells were plated in a 6-well plate and grown with and without formate. Cells were enumerated at 48 hours; no significant difference in Henle-407 cells per monolayer was observed. Download FIG S3, TIF file, 1.83 MB. Copyright © 2018 Koestler et al. 2018 Koestler et al. This content is distributed under the terms of the Creative Commons Attribution 4.0 International license.

Listeria monocytogenes is another pathogen that, similarly to *Shigella*, accesses the host cell cytosol and exploits host actin polymerization for cell-to-cell spread. We postulated that if formate was affecting host cell physiology to promote bacterial cell-to-cell spread, it would increase the plaque size of L. monocytogenes, as well as *Shigella*. However, supplementation with exogenous formate had no significant effect on the plaque size of L. monocytogenes in Henle-407 cell monolayers (Fig. S5), further supporting the hypothesis that enhanced spread of intracellular S. flexneri in the presence of formate is a response by the bacteria, rather than the host cell.

The role of formate in increasing *Shigella* plaque size could be due to faster growth of the bacteria or increased spread. There was no significant difference in S. flexneri intracellular doubling time between the presence and absence of exogenous formate (Fig. 1D). However, we observed an increase in S. flexneri cell-to-cell spread in the presence of exogenous formate (Fig. 1E), suggesting that formate promotes S. flexneri cell-to-cell spread, resulting in increased plaque size in Henle-407 monolayers.

S. flexneri has only one known formate transporter, FocA. FocA is a bidirectional formate transporter that directly interacts with PFL to export formate (27–29). FocA activity is dependent on pH, and when the pH is below 5.8, the function of FocA switches to a formate importer (28). *focA* is cotranscribed with *pflB* and expressed under aerobic or microaerobic conditions under the control of ArcA and the fumarate and nitrate reduction regulator FNR (30–32). We found that mutating S. flexneri *focA* reduced plaque size similarly to the *ΔpflB* mutant, likely due to reduced formate secretion. However, plaque size of the *ΔfocA* strain increased with exogenous formate (Fig. S4), indicating that S. flexneri formate import via FocA is dispensable for formate promotion of plaque size and suggesting that formate is sensed by S. flexneri outside the bacterial cytoplasm or, less probably, that formate is imported through an unidentified transporter.

10.1128/mBio.01777-18.4 FIG S4 Plaque size of S. flexneri WT, Δ*pflB*, and Δ*focA* strains was measured; loss of *focA*, which transports formate, decreases plaque size similarly to the Δ*pflB* strain; however, formate significantly increases Δ*focA* plaque size. An asterisk indicates statistical significance. Download FIG S4, TIF file, 1.58 MB. Copyright © 2018 Koestler et al. 2018 Koestler et al. This content is distributed under the terms of the Creative Commons Attribution 4.0 International license.

10.1128/mBio.01777-18.5 FIG S5 Plaque size of Listeria monocytogenes was measured; formate has no significant effect on L. monocytogenes plaque size. Giemsa stains of plaques underneath corresponding measurements. Download FIG S5, TIF file, 3.50 MB. Copyright © 2018 Koestler et al. 2018 Koestler et al. This content is distributed under the terms of the Creative Commons Attribution 4.0 International license.

### *pflB* is required for S. flexneri formate secretion.

**FIG 2:**
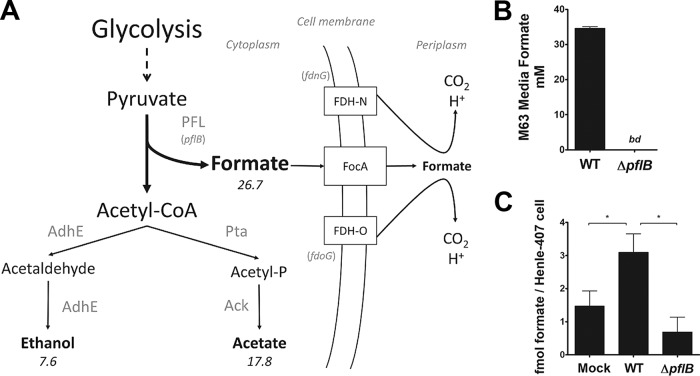
(A) Model of S. flexneri fermentative metabolism. S. flexneri metabolism was simulated using a genome-scale metabolic network model; numbers indicate relative carbon flux. Under anoxic conditions, S. flexneri is predicted to secrete formate, acetate, and ethanol at a ratio of approximately 3:2:1. Formate is a by-product of PFL-mediated conversion of pyruvate to acetyl-CoA. (B) S. flexneri*-*secreted formate was measured in the supernatant from bacteria grown anaerobically in M63 medium. *bd* indicates “below detection.” No formate secretion was observed in the *ΔpflB* mutant. (C) Intracellular formate was measured in uninfected and infected Henle-407 cells at 3 hpi. Formate was normalized to the total number of Henle-407 cells. WT S. flexneri infection increased intracellular formate, while infection with the Δ*pflB* mutant did not.

While S. flexneri metabolism is understudied, metabolism and mixed acid fermentation of the closely related bacterium E. coli have been characterized in detail, and the predominant E. coli fermentation products *in vitro* are formate, acetate, ethanol, and lactate (33). In limited oxygen, E. coli metabolizes carbon using glycolysis, producing pyruvate for mixed acid fermentation. Pyruvate is converted to lactate via lactate dehydrogenase or to acetyl-CoA via pyruvate dehydrogenase (PDH) or PFL; of these two enzyme complexes which generate acetyl-CoA, only PFL produces formate as a by-product. Acetyl-CoA is then converted to acetate or ethanol, while formate is predominantly secreted through one of two bidirectional transporters, FocA or FocB (28, 34). Cytoplasmic formate can be oxidized by the formate hydrogenlyase complex (FHL), while periplasmic formate can be oxidized by one of two formate dehydrogenase complexes (FDH-N or FDH-O). The genomes of E. coli and S. flexneri are highly similar, allowing us to project S. flexneri mixed acid fermentation (summarized in Fig. 2A). Of note, the S. flexneri locus containing *focA* and the PFL genes (including *pflB*) is highly conserved among sequenced *Shigella* species (Fig. S6). However, there are other notable differences in metabolism genes between E. coli and S. flexneri; S. flexneri has 58 metabolism-related pseudogenes compared to E. coli, including the putative formate transporter *focB*, and *fdhF*, a gene essential for the activity of FHL (35). We simulated S. flexneri metabolism *in silico* using a published S. flexneri genome-scale metabolic model (36, 37), which integrates S. flexneri genome data and enzyme stoichiometry of metabolic reactions into a metabolic network; constraint-based analysis is then applied to emulate metabolism maximizing biomass production (38, 39). To mimic intracellular conditions, we simulated a low-oxygen environment where carbon availability limits total growth. Under these conditions, S. flexneri is predicted to produce formate, acetate, and ethanol *in silico* at a ratio of approximately 3:2:1 and no lactate (Fig. 2A). The model predicted that 29.7% of total carbon available is converted to formate under the simulated conditions. This estimation is consistent with previous studies examining mixed acid fermentation in S. flexneri and E. coli (16, 40).

10.1128/mBio.01777-18.6 FIG S6 (A) Schematic of the PFL locus of S. flexneri 2457T. (B) The PFL loci from 79 Shigella genomes were aligned, and phylogenetic analysis reveals that the PFL locus of S. flexneri 2457T is highly conserved from E. coli and among Shigella spp. S. flexneri 2457T is highlighted in green, while E. coli MG1655 is highlighted in blue. Other gastrointestinal pathogens are included for comparison. Download FIG S6, TIF file, 1.88 MB. Copyright © 2018 Koestler et al. 2018 Koestler et al. This content is distributed under the terms of the Creative Commons Attribution 4.0 International license.

The amount of formate secreted by the S. flexneri WT and Δ*pflB* mutant was quantified from cells grown in minimal medium, under the conditions simulated in the *in silico* model. After 18 h of growth, the concentration of formate secreted by the WT S. flexneri strain was 34.6 ± 0.5 mM, translating to a 38.4% conversion of available carbon to formate (Fig. 2B). In contrast, there was no detectable formate in supernatants from the Δ*pflB* mutant, confirming that PFL is essential for S. flexneri formate production. At host cytosolic pH, formate exists as a monovalent anion that cannot passively diffuse across bacterial or eukaryotic cellular membranes, with the exception of acidic compartments such as lysosomes (21); we therefore postulated that host cytosolic formate concentration would increase during S. flexneri infection due to S. flexneri metabolism and the spatial restriction imposed by the host cell membrane. We observed a 2.1-fold increase in intracellular formate of infected cells compared to uninfected cells (Fig. 2C). In contrast, Henle-407 cells infected with the S. flexneri *ΔpflB* mutant showed no increase in intracellular formate concentration.

### FDH-N inhibits S. flexneri plaque formation.

**FIG 3:**
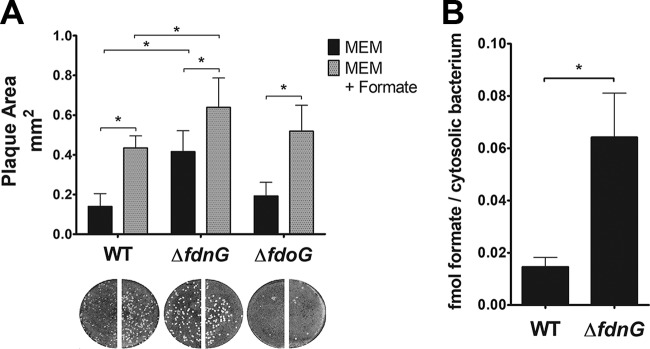
(A) The Δ*fdnG* mutant forms larger plaques. Plaque size of S. flexneri WT, FDH-N mutant (Δ*fdnG*), or FDH-O mutant (Δ*fdoG*) was measured; loss of FDH-N, which oxidizes formate to CO_2_, increased plaque size. (B) Formate accumulates to higher levels in Henle-407 cells infected with the Δ*fdnG* mutant. Formate was normalized to the number of intracellular bacteria. An asterisk indicates statistical significance.

S. flexneri encodes two periplasm-facing molybdoselenoformate dehydrogenase complexes, FDH-N and FDH-O, which couple formate oxidation to nitrate reduction. E. coli FDH-N, which catalyzes the conversion of periplasmic formate to H^+^ and CO_2_, is expressed during anaerobic growth and induced by nitrate in a NarL-dependent manner (41); FDH-O is active in the presence of oxygen and accounts for a smaller portion of the total formate dehydrogenase activity (42, 43). While S. flexneri PFL and FDH-N levels are elevated within an epithelial cell, expression of FDH-O-related genes is repressed (5, 15), suggesting that FDH-N is more important than FDH-O for formate metabolism by intracellular S. flexneri. If S. flexneri FDH-N is responsible for formate catabolism during intracellular growth, deletion of FDH-N would increase intracellular formate in infected Henle-407 cells and thus increase plaque size. We examined the plaque formation of S. flexneri Δ*fdnG* (catalytic subunit of FDH-N) and Δ*fdoG* (catalytic subunit of FDH-O) mutants. There was a 3.0-fold increase in plaque size of the S. flexneri Δ*fdnG* strain over WT (Fig. 3A). Furthermore, in the presence of exogenous formate the plaque size of the Δ*fdnG* strain increased 1.5-fold over the WT with formate. We measured formate levels of Henle-407 cells infected with S. flexneri WT and the Δ*fdnG* strain and found that formate was significantly higher in the Δ*fdnG* strain than the WT strain, consistent with reduced S. flexneri formate oxidation (Fig. 3B). These data indicate that FDH-N reduces S. flexneri spread, presumably by oxidizing formate derived from either S. flexneri or host metabolism and decreasing its concentration within the host cell cytosol. In contrast, the plaque size of the Δ*fdoG* strain was not significantly different from that of the WT strain, regardless of exogenous formate. This is consistent with the low expression of *fdoG* in intracellular bacteria (14). However, the Δ*fdoG* strain formed significantly fewer plaques than the WT strain, indicating that FDH-O, which is more highly expressed extracellularly, may be important for efficient invasion of host cells.

### Formate alters S. flexneri virulence gene expression.

To identify S. flexneri genes affected by formate that could promote plaque formation, we determined formate-induced differences in the S. flexneri transcriptome using RNA sequencing (RNA-seq). We were able to map an average of 3 × 10^6^ unique reads per sample to the S. flexneri genome, and we found that exogenous formate significantly altered the expression of four genes on the virulence plasmid (Table 1). The S. flexneri gene *icsA*, which recruits host N-WASP for actin-mediated intercellular spread, was upregulated 2.1-fold by formate. Additionally, two S. flexneri T3SS effectors were upregulated approximately 2-fold by formate: *ipaJ* and *ipgD*. Formate also altered the expression of the virulence plasmid maintenance gene *ccdB* and 13 S. flexneri chromosomal genes (Table 1); however, we focused on *icsA*, *ipaJ*, and *ipgD*, since these genes are known virulence determinants.

**TABLE 1:** *S. flexneri* genes differentially regulated during infection of Henle-407 cells with exogenous 20 mM formate

Carriage location and gene	Function	Fold change
Virulence plasmid		
*ipaJ*	Host protein N-myristoylation	2.3
*icsA*/*virG*	Actin-mediated intercellular spread	2.1
*ipgD*	Host phosphoinositide metabolism	2.1
*ccdB*	Plasmid maintenance	−2.1
Chromosome		
*yjhA*	Hypothetical protein	3.3
*yccJ*	Hypothetical protein	2.5
*yhaH*	Putative cytochrome	2.3
*rfbE*	Polysaccharide biosynthesis	2.3
*melR*	Regulator of melibiose operon	2.1
*ybeK*	Putative tRNA synthetase	2.1
*yjhT*	Hypothetical protein	2.1
*yhiO*	Hypothetical protein	2.0
*proV*	Osmotic regulation	−2.1
*Rnd*	RNase D	−2.2
*S2640*	Hypothetical protein	−2.3
*yebJ*	Hypothetical protein	−2.4
*ybaN*	Hypothetical protein	−2.6

**FIG 4:**
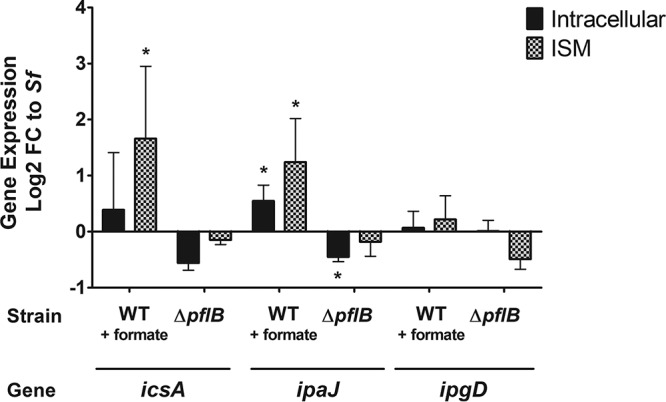
Formate increases the expression of *icsA* and *ipaJ. S. flexneri icsA*, *ipaJ*, and *ipgD* expression in Henle-407 cells at 3 hpi (black bars) or ISM (checkered bars) was independently determined by RT-qPCR. Log_2_ fold change is relative to WT S. flexneri. An asterisk indicates statistical significance.

To validate the findings of our RNA-seq, we quantified S. flexneri *icsA*, *ipaJ*, and *ipgD* transcript levels during growth *in vitro* or in Henle-407 cells using quantitative reverse transcriptase PCR (RT-qPCR). We observed significant sample-to-sample variation both *in vitro* and *in vivo*; however, formate supplementation significantly increased S. flexneri *icsA* and *ipaJ in vitro* (Fig. 4). We also observed that exogenous formate increased intracellular S. flexneri *ipaJ* expression 1.5-fold, while the Δ*pflB* mutant showed a 1.3-fold decrease in *ipaJ* expression compared to WT (Fig. 4). Both exogenous formate and *pflB* had no significant impact on S. flexneri *ipgD* levels either *in vitro* or in Henle-407 cells (Fig. 4).

### Formate increases surface S. flexneri IcsA.

**FIG 5:**
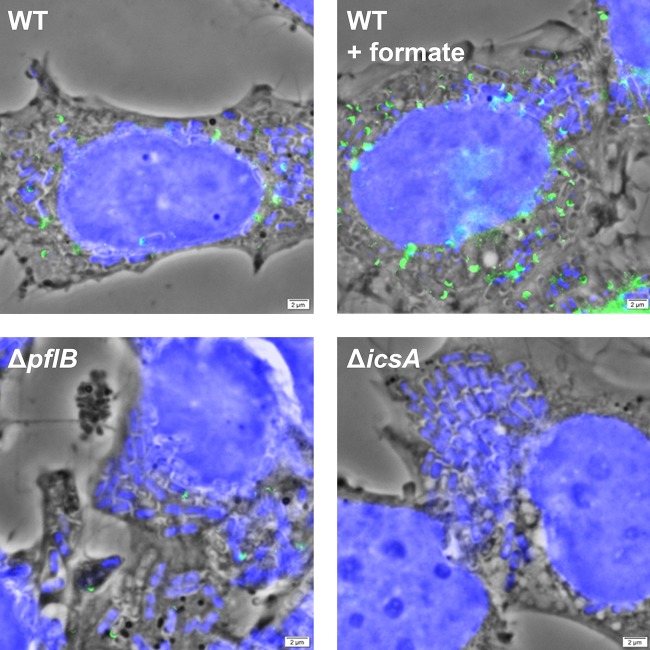
Formate increases S. flexneri IcsA in infected Henle-407 cells. Polarly localized IcsA (green) was visualized in intracellular S. flexneri (blue) using fluorescence microscopy. Fluorescence images were overlaid on a phase-contrast micrograph. We observed more polar IcsA WT S. flexneri grown with formate and lower polar IcsA levels in the *ΔpflB* mutant than in the WT strain.

S. flexneri uses polarly localized IcsA to recruit host N-WASP, catalyzing host actin synthesis and propelling the bacterium through the host cell cytosol (44, 45). Δ*icsA* mutants cannot spread and do not form plaques in Henle-407 monolayers (2, 46); therefore, to confirm that S. flexneri *icsA* is differentially regulated by formate, S. flexneri IcsA was visualized in infected Henle-407 cells by fluorescence staining and microscopy. IcsA labeled with fluorescein isothiocyanate (FITC) appeared as green, U-shaped foci on the micrographs that corresponded with the poles of intracellular bacteria, which were stained blue with 4′,6-diamidino-2-phenylindole (DAPI) (Fig. 5). As expected, no green foci were observed in cells infected with the Δ*icsA* mutant. We observed more S. flexneri with IcsA staining (45.8% ± 0.5%) in infected Henle-407 cells supplemented with formate than in those without formate supplementation (22.0% ± 6%), and the foci appeared brighter. In contrast, a smaller percentage of the intracellular S. flexneri Δ*pflB* mutant showed IcsA staining (7.0% ± 1.7%), and the foci were dimmer than with the WT strain. These findings together suggest that formate increases the levels of S. flexneri IcsA in infected Henle-407 cells.

### *ipgD* is required for formate-mediated plaque formation.

**FIG 6:**
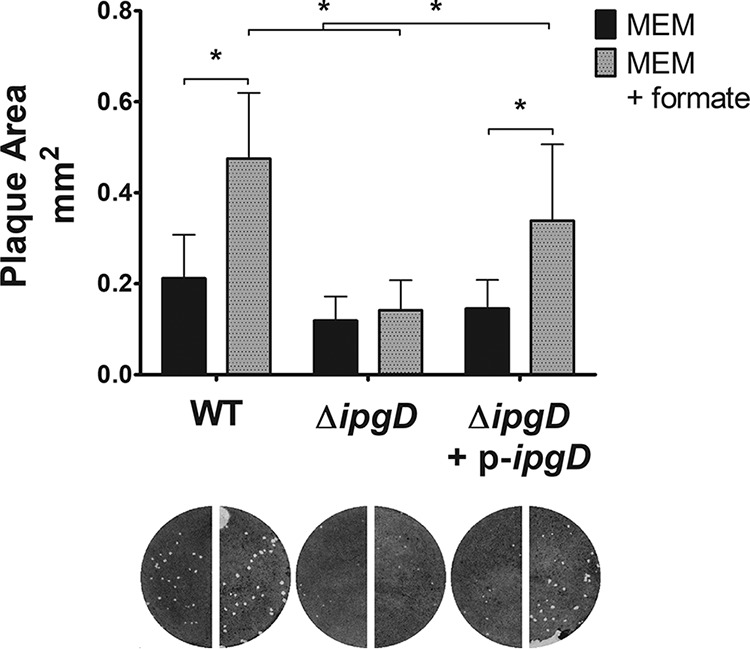
The *ipgD* mutant does not increase plaque size with formate. Plaque size of S. flexneri WT and *ΔipgD* mutant was measured in cultured cells in the presence or absence of exogenous formate. (An asterisk indicates statistical significance.) The *ΔipgD* mutant did not increase plaque size in the presence of exogenous formate, and this phenotype is complemented by providing *ipgD* on a plasmid.

S. flexneri IpgD is a secreted PtdIns(4,5)P_2_ 4-phosphatase that produces PtdIns5P in the host cell; increased PtdIns5P has widespread effects on the host, including alteration of host membrane tension, activation of host Akt kinase, inhibition of T-cell migration, inhibition of host extracellular ATP (eATP) secretion, activation of host ARF6, and altered host Ca^2+^ signaling (8, 47–50). To determine if *ipgD* is involved in the S. flexneri response to formate, we measured the plaque size of an S. flexneri *ΔipgD* mutant in Henle-407 monolayers with exogenous formate. Compared to the S. flexneri WT strain, the *ΔipgD* mutant formed smaller plaques (Fig. 6). Importantly, the *ΔipgD* mutant showed no significant increase in plaque size when the tissue culture medium was supplemented with 20 mM formate. When S. flexneri *ipgD* was complemented on a plasmid, the formate-mediated increase in plaque size was restored (Fig. 6). This suggests that *ipgD* is required for the formate-mediated increase in S. flexneri plaque size.

### *ipaJ* contributes to plaque size increase by formate.

**FIG 7:**
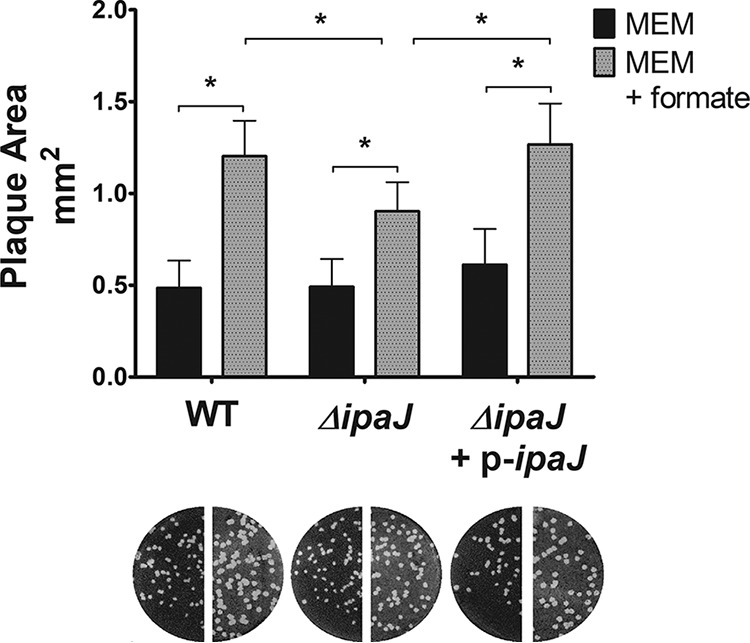
The *ipaJ* mutant has reduced response to formate. Plaque size of S. flexneri WT and *ΔipaJ* mutant was measured in cultured cells in the presence or absence of exogenous formate. (An asterisk indicates statistical significance.) The difference in plaque size caused by the addition of exogenous formate is significantly less in the *ΔipaJ* mutant than in the WT strain, and this phenotype is complemented by providing *ipaJ* on a plasmid.

IpaJ is an S. flexneri*-*secreted cysteine protease that disrupts the host Golgi apparatus by altering host protein N-myristoylation (11, 12, 51). We examined how the plaque size of an S. flexneri Δ*ipaJ* mutant changes in response to exogenous formate. We found that in the absence of exogenous formate, the mean plaque size of the Δ*ipaJ* mutant was not significantly different from that of the WT strain (Fig. 7), consistent with previous reports (52). The Δ*ipaJ* mutant response to formate was diminished compared to WT, with the mutant forming smaller plaques than the WT strain when both were supplemented with exogenous formate; this phenotype was complemented by providing *ipaJ* on a plasmid (Fig. 7). These results indicate that *ipaJ* contributes to the formate-mediated increase in S. flexneri plaque size.

### *ipaJ* expression is dependent on S. flexneri cell density and formate.

**FIG 8:**
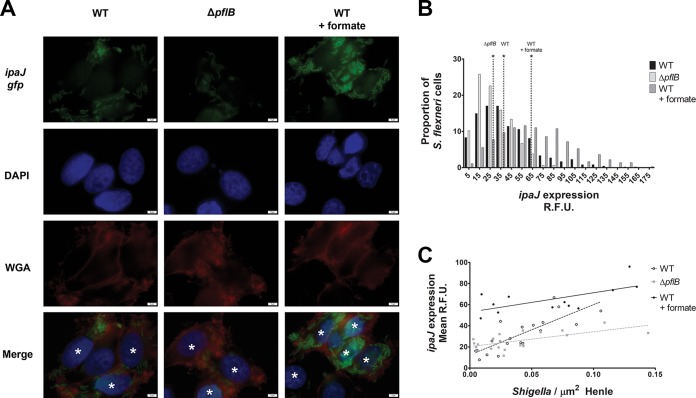
Expression of *ipaJ* in the intracellular environment increases in the presence of formate and is influenced by bacterial cell density and formate concentration in the Henle-407 cells. (A) The *ipaJ* promoter was fused to *gfp* (green), and infected Henle-407 cells were visualized using fluorescence microscopy. Nuclei were stained with DAPI (blue), and Henle-407 membranes were stained with wheat germ agglutinin (red). Asterisks indicate infected cells. The Δ*pflB* mutant displayed lower *gfp* intensity, while formate increased *gfp* intensity in WT. (B) *ipaJ* expression was measured by quantitative fluorescence microscopy. Dotted lines indicate median *gfp* intensity. An asterisk indicates statistical significance compared to each of the other two medians. Formate increased *ipaJ* expression 1.8-fold. (C) *ipaJ* expression of intracellular S. flexneri was regressed on S. flexneri density. The slope of WT S. flexneri *ipaJ* expression (*R*^2^ = 0.7) was significantly different from that of WT plus formate (*R*^2^ = 0.4) and the Δ*pflB* strain (*R*^2^ = 0.4).

Because S. flexneri secretes formate in the host cytosol as a by-product of carbon metabolism, and this formate accumulates in the host cytosol of S. flexneri-infected Henle-407 cells (Fig. 2C), we expected that formate-induced virulence gene expression would increase as S. flexneri intracellular density increased, as formate is produced from bacterial metabolism. To test this, we constructed an *ipaJ* transcriptional reporter, in which the promoter region of *ipaJ* was fused to *gfp* on a plasmid, and *ipaJ* expression was visualized using fluorescence microscopy. We observed increased fluorescence in the intracellular S. flexneri WT cells compared to the Δ*pflB* mutant (Fig. 8A); likewise, formate supplementation of Henle-407 cells infected with WT S. flexneri increased fluorescence. Green fluorescent protein (GFP) fluorescence was then measured in single bacterial cells from fluorescence micrographs. We observed a 1.8-fold increase in *gfp* of S. flexneri when exogenous formate was added as a supplement (Fig. 8B), similar to the changes in *ipaJ* levels observed in previous experiments (Table 1 and Fig. 4). Likewise, we observed a 1.3-fold decrease of *ipaJ* expression in the Δ*pflB* mutant compared to the WT strain. We then measured the number of intracellular S. flexneri bacteria and the two-dimensional cell area of Henle-407 cells stained with wheat germ agglutinin (WGA) and regressed mean *ipaJ* expression of S. flexneri within individual Henle-407 cells on intracellular S. flexneri density. *ipaJ* expression positively correlated with S. flexneri intracellular density (Fig. 8C), and when cells were supplemented with exogenous formate, *ipaJ* expression levels were constitutively elevated regardless of intracellular S. flexneri density. Furthermore, *ipaJ* expression of the *ΔpflB* mutant was lower than that of the WT strain, regardless of intracellular S. flexneri density. These data indicate that intracellular *ipaJ* expression is dependent on the intracellular density of S. flexneri cells, and this regulation is positively associated with formate accumulation.

### Altering formate results in modified host response of S. flexneri*-*infected Henle-407 cells.

**FIG 9:**
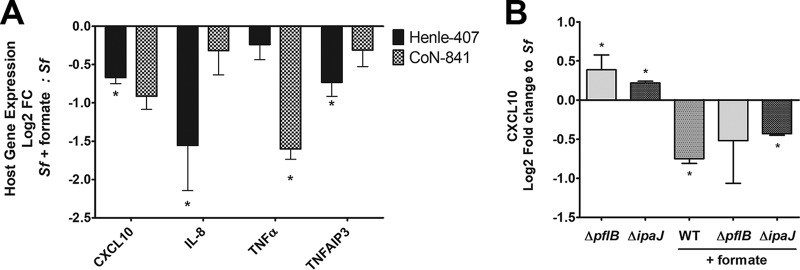
Formate inhibited interferon- and TNF-stimulated gene expression levels in infected Henle-407 and CoN-841 cells. (A) Host gene expression of infected Henle-407 cells (black bars) and CoN-841 cells (gray checkered bars) was determined by RT-qPCR. (B) CXCL10 transcript levels of Henle-407 cells infected with different S. flexneri strains were determined by RT-qPCR. Log_2_ fold change is relative to host cells infected with WT S. flexneri in the absence of exogenous formate, and an asterisk indicates statistical significance compared to WT infection.

S. flexneri infection elicits a strong inflammation response from the host cell (8, 53). To counteract this, S. flexneri employs IpaJ and IpgD, which dampen host interferon- and tumor necrosis factor (TNF)-stimulated genes (8, 11, 12). Because *ipgD* and *ipaJ* contribute to the S. flexneri response to formate (Fig. 6 and 7), we hypothesized that supplementation with exogenous formate would result in altered host immune response to S. flexneri infection. We selected 4 host genes known to be affected by either *ipgD* or *ipaJ*: CXCL10, interleukin-8 (IL-8), TNF-α, and TNFAIP3 (8, 11). We found that exogenous formate lowered the levels of CXCL10, IL-8, and TNFAIP3 in S. flexneri-infected Henle-407 cells (Fig. 9A, black bars), while in uninfected cells, exogenous formate had no effect on CXCL10, IL-8, TNF-α, or TNFAIP3 expression (1.0-, 1.1-, 0.9-, and 1.1-fold change, respectively).

Henle-407 cells, a HeLa derivative cell line, have altered immune pathways relating to S. flexneri infection response. For example, Henle-407 cells do not express the connexin hemichannels involved in secretion of eATP, a potent inflammatory signal (54). Therefore, we analyzed S. flexneri*-*infected CoN-841 cells grown with or without exogenous formate and quantified CXCL10, IL-8, TNF-α, or TNFAIP3 expression levels. We observed more gene expression variation in infected CoN-841 cells than in Henle-407 cells, and TNF-α was the most strongly repressed gene in infected CoN-841 cells supplemented with exogenous formate (Fig. 9A, checkered bars), suggesting that the specific genes and the dynamics of the host immune response in regard to formate-regulated S. flexneri effectors vary in a different human cell line.

To confirm the effect of formate on S. flexneri *ipaJ*, we examined host CXCL10 expression in Henle-407 cells infected with the Δ*pflB* and Δ*ipaJ* mutant strains. In contrast to formate lowering CXCL10 expression in cells infected with WT S. flexneri, CXCL10 expression was increased in Henle-407 cells infected with the S. flexneri Δ*pflB* mutant, and this difference was abated when exogenous formate was provided as a supplement (Fig. 9B). Similarly to the Δ*pflB* mutant, CXCL10 expression was elevated in Henle-407 cells infected with the S. flexneri Δ*ipaJ* mutant, and formate reduced this response.

## DISCUSSION

Formate is an important biomolecule for both human and bacterial metabolism. In bacteria, formate is a by-product of pyruvate formate lyase (PFL)-mediated conversion of pyruvate to acetyl-CoA. In addition to PFL, *Shigella* encodes a pyruvate dehydrogenase (PDH) complex that converts pyruvate to acetyl-CoA under aerobic conditions but does not produce formate. An S. flexneri Δ*aceE* mutant defective in PDH has an intracellular growth defect, indicating that PDH is utilized for acetyl-CoA generation within the host cell (16). Eukaryotic cells contain low levels of cytosolic oxygen, a condition that permits the activity of both PFL and PDH, which explains why the S. flexneri Δ*pflB* mutant intracellular growth rate is unaffected despite missing this source of acetyl-CoA (16).

Eukaryotic cells metabolize formate via one-carbon metabolism for purine biosynthesis, among other things (55). At biological pH, formate cannot passively cross bacterial or eukaryotic cell membranes (21); however, extracellular formate exchange occurs in cultured human cells (56–58), presumably facilitated through the formate transporter SLC26A6 (56, 59, 60). Likewise, formate is shuttled between the mitochondria and eukaryotic cytosol through an unknown mechanism (55). It is unclear how Henle-407 cells react to the influx of cytosolic formate from S. flexneri infection. We predict that a portion of S. flexneri-derived host cytosolic formate is oxidized by S. flexneri FDH-N (Fig. 3), a second portion is converted to purines by host one-carbon metabolism, and a third portion accumulates in the host cytosol.

Formate turnover has been previously linked to the intracellular growth phase of S. flexneri (5, 15). Here, we demonstrate that it is not the metabolism of formate but formate itself that promotes S. flexneri plaque formation by increasing intercellular spread via IcsA and modulating the host response to S. flexneri infection. While PFL can be reversed to convert formate to pyruvate for metabolism (61), formate increases Δ*pflB* mutant plaque size, indicating that formate is not used solely as a substrate for PFL to produce pyruvate for growth (Fig. 2B). And, while formate oxidation is a potential source of NADH, we also show that formate oxidation is dispensable for intracellular S. flexneri metabolism, since exogenous formate does not increase S. flexneri growth rate within the host cell (Fig. 1C) and knocking out FDH-N promotes S. flexneri plaque formation (Fig. 4). Unlike the closely related E. coli, *Shigella* spp. (including S. flexneri) have a mutated FHL, suggesting an evolutionary selection against *Shigella* formate oxidation (35); however, formate dehydrogenases contribute to the fitness of closely related E. coli in the lumen during inflammation-associated colon dysbiosis (62), possibly explaining why S. flexneri maintains its formate dehydrogenases.

Although it is clear that extracellular formate can enter Henle-407 cells when provided as an exogenous supplement, as shown by uptake of radiolabeled formate (see Fig. S2 in the supplemental material) and alterations in S. flexneri gene expression (Table 1; Fig. 4 and 8), we believe that *Shigella-*derived formate is sufficient to differentially regulate expression related to intercellular spread and host immune dampening because we observe these phenotypes in S. flexneri formate metabolism mutants. We propose a model in which, following entry into the host cell cytoplasm, S. flexneri begins to replicate and metabolize host sugars and pyruvate, producing and secreting formate as a by-product. Formate accumulates in the infected cell cytosol rapidly due to the spatial constrictions of the host cell membrane and induces *icsA* to promote S. flexneri intercellular spread. Formate also induces the expression of *ipaJ* and possibly *ipgD* to alter the host immune response to S. flexneri infection. Because formate regulates S. flexneri *icsA* and *ipaJ* expression *in vitro* (Fig. 4), S. flexneri likely possesses a formate sensory mechanism; this S. flexneri formate sensor would likely act at the outer membrane or periplasm, as formate increases plaque size of the *ΔfocA* formate transport mutant (Fig. S2). One possible sensing mechanism is the BarA-UvrY two-component regulatory system, which senses formate and short-chain fatty acids to modulate the activity of the Csr regulatory system in E. coli (63) and regulates virulence in S. flexneri and Salmonella enterica (25, 64). However, the fact that acetate fails to increase plaque size indicates that BarA-UvrY is not the sensory mechanism; consistent with this, we found that S. flexneri Δ*barA* or Δ*uvrY* mutants still increase plaque size in response to formate (Fig. S5).

Previous studies demonstrate that there is spatiotemporal regulation of S. flexneri*-*secreted effectors during host cell infection (5, 6, 65–67). Our data indicate that formate is an S. flexneri signal to differentially regulate *icsA* and *ipaJ* expression in the host cell, and this regulation is correlated with increased intracellular S. flexneri density. The dynamic nature of formate-mediated S. flexneri virulence gene regulation could explain the variation in both host and S. flexneri gene expression that we observed in response to formate (Fig. 4 and 9) and why there were inconsistencies such as formate-altered S*. flexneri ipgD* expression (Table 1 and Fig. 4). The production of formate is not essential to S. flexneri virulence, evidenced by the fact that the Δ*pflB* mutant is still able to form plaques, albeit smaller than those of the WT. Rather, formate-mediated regulation of *icsA/ipaJ* appears to be a way to fine-tune expression in response to intracellular bacterial density or to facilitate spread once a threshold density has been achieved. There is currently a high degree of interest in the complex relationship between metabolism and pathogenesis (68, 69), and the concept of a metabolic by-product regulating bacterial virulence is not new; one example is indole, a by-product of tryptophan hydrolysis that regulates virulence phenotypes and has been reported as a social signal in enteric microbial communities (70–72). It is easy to draw comparisons between indole and formate signaling; however, one notable difference between indole and formate signaling is that indole is diffusible across cellular membranes while formate is not (73), allowing cytosolic formate to accumulate during intracellular infection.

As the bacterial load within a host cell increases, so does the need of the bacteria to dampen the host immune response in order to evade host cytosolic defenses such as autophagy, as well as systemic defenses such as neutrophil recruitment and inflammation. Consistent with other pathogens that suppress host immune response, immunomodulating effectors secreted by a single S. flexneri cell, which are costly to produce, benefit the entire intracellular S. flexneri population (74). Thus, we consider modulating host response to be a cooperative social behavior which conveys a benefit to S. flexneri intracellular populations. If formate sensing is social in nature, it could convey local information about both intracellular S. flexneri cell density and S. flexneri spatial constraint, as formate accumulation differs in the intracellular and extracellular environments. It should also be noted that inflammation in the colon results in higher levels of formate in the lumen (59). During *Shigella* infection, the *Shigella*-induced inflammatory response could lead to increased formate in the lumen and subsequently in the epithelial cells, promoting bacterial spread and production of anti-inflammatory effectors. A more complete understanding of the role of formate production and sensing in infected host cells will provide a better understanding of this complex host-pathogen interaction.

## MATERIALS AND METHODS

### Media and growth conditions.

Bacterial strains, plasmids, and primers used in this study are listed in Table S1 in the supplemental material. *S. flexner*i was cultured on tryptic soy broth agar plates with 0.01% (wt/vol) Congo red (TSBA-CR), and red colonies were selected to ensure the presence of the S. flexneri virulence plasmid. Overnight bacterial cultures were grown in Luria-Bertani (LB) broth at 30°C, subcultured 1:100, and grown at 37°C to an optical density at 650 nm (OD_650_) of 0.5 to 1.0 (mid-log phase) prior to infection. Deoxycholate (DOC) was added as a supplement at 0.1% (wt/vol) where indicated to increase efficiency of invasion of Henle-407 cultured cells (75). Antibiotics were added as supplements where indicated at the following concentrations: 25 μg/ml ampicillin, 50 μg/ml kanamycin, 6 μg/ml chloramphenicol, and 20 μg/ml gentamicin. Sodium formate was added as a supplement where indicated at 20 mM.

10.1128/mBio.01777-18.8 TABLE S1 (A) Human tissue culture lines and bacterial strains used in this study, along with their respective sources. (B) Bacterial plasmids used in this study. (C) Oligonucleotide sequences of primers for cloning, sequencing, and RT-qPCR used in this study. Download Table S1, DOCX file, 0.02 MB. Copyright © 2018 Koestler et al. 2018 Koestler et al. This content is distributed under the terms of the Creative Commons Attribution 4.0 International license.

Henle-407 cells (ATCC intestinal 407, CCL-6) and CoN-841 cells (ATCC CCD 841, CRL-1790) were cultured in minimal essential medium (MEM; Gibco 61100-087) supplemented with 10% (vol/vol) Bacto tryptone phosphate broth (Difco), 10% (vol/vol) fetal bovine serum (Gibco 16140-071), 2 mM glutamine, and nonessential amino acids (Gibco 11140-050). Henle-407 and CoN-841 cells were grown at 37°C with 5% CO_2_.

### Construction of S. flexneri mutants and plasmids.

Strains, primers, and plasmids used in this study are listed in Table S1. The Δ*fdnG*, Δ*fdoG,* and Δ*focA* mutants were created using P1 bacteriophage transduction of the genes from E. coli strains JW1470, JW3865, and JW0887 (respectively) from the Keio collection (76). The Δ*ipaJ* mutant was generated using a modified method of Datsenko and Wanner (77, 78). Overlap extension PCR was used to fuse 399 bp upstream and 383 bp downstream of *ipaJ* to each side of the chloramphenicol resistance cassette of pKD3 (77). This product was amplified by PCR using the ipaJKO-1 and ipaJKO-4 primers, ethanol precipitated, and brought to a concentration of >500 ng/μl in water. S. flexneri containing the plasmid pKD46 (77) was grown to mid-log phase in 25 ml LB with no NaCl at 30°C, and then 2 mM arabinose was added and the culture was brought to 37°C for 30 min. Cells were pelleted and resuspended in 500 μl warm water, mixed with 10 μl *ipaJ* knockout (KO) PCR product, and immediately electroporated. Mutants were selected on TSBA-CR with chloramphenicol. All mutants were verified by PCR and DNA sequencing at the University of Texas at Austin DNA sequencing facility.

The *ipaJ* complement plasmid (pBK24) was constructed by amplifying the *ipaJ* locus beginning ∼400 bp upstream of the annotated *ipaJ* gene and ending at the *ipaJ* stop codon from S. flexneri DNA by PCR with primers ipaJ-30c-fw and ipaJ-30c-rv, containing 5′ KpnI and XbaI sites, respectively. After restriction digest, the product was ligated into the corresponding sites of the plasmid pWKS30 (79). The *ipaJ-gfp* transcriptional reporter plasmid (pBK25) was constructed by amplifying the *ipaJ* promoter region, beginning ∼500 bp upstream of *ipaJ* and ending at the *ipaJ* start codon, by PCR using primers ipaJ-gfp-fw and ipaJ-gfp-rv containing 5′ SmaI and XbaI sites. After restriction digest, the product was ligated into the corresponding sites of the plasmid pLR29 (14). Both plasmids were confirmed by PCR and DNA sequencing at the University of Texas at Austin DNA sequencing facility.

### *In silico* analysis of S. flexneri metabolism.

*In silico* metabolism simulations were performed using a previously published S. flexneri genome-scale metabolic network reconstruction (36) and OptFlux (37). We removed external boundary metabolites and used the core biomass production as our objective function and the pFBA simulation method. To simulate anaerobic growth in M63 medium, default environmental conditions were used, except that the lower bound of O_2_ exchange was set to 0, the lower bound of nicotinate exchange was set to −0.162, and the lower bounds of glucose and pyruvate exchange were set to −10. Total biomass production was limited by carbon availability under these simulated conditions.

### Tissue culture assays.

Plaque assays were performed as described previously (18) with modifications (17). Bacteria at mid-log growth were centrifuged and resuspended in sterile saline to a final concentration of 5 × 10^4^ CFU/ml, and 100 μl of this suspension was added to Henle-407 monolayers grown to confluence. Plates were centrifuged for 10 min at 1,000 × *g* and incubated for 60 min at 37°C and 5% CO_2_. Monolayers were then washed four times with phosphate-buffered saline (PBS) and incubated in MEM supplemented with 0.2% glucose, gentamicin, and formate where indicated for either 48 or 72 h. Monolayers were stained with Wright-Giemsa stain (Camco) and imaged using an Alpha Innotech AlphaImager (Protein Simple), and plaque area was measured using ImageJ (80). Statistical significance was determined by analysis of variance (ANOVA) with a Bonferroni posttest, *P* < 0.05 (GraphPad Prism); figures are representative of at least 2 independent experiments.

For L. monocytogenes plaque assays, L. monocytogenes was grown in brain heart infusion (BHI) broth for approximately 18 h at 30°C without shaking. One milliliter of culture was centrifuged at 13,000 × *g* for 2 min, and then the supernatant was removed and the pellet was resuspended in 1 ml PBS. Two microliters of L. monocytogenes suspension was added to Henle-407 monolayers grown in a 6-well plate and gently shaken for 1 min. The plate was incubated for 1 h at 37°C and 5% CO_2_. The monolayers were then washed four times with PBS and incubated in MEM supplemented with 0.2% glucose, gentamicin (10 μg/ml), and formate where indicated for 48 to 72 h. Monolayers were stained with Wright-Giemsa stain (Camco) and imaged using an Alpha Innotech AlphaImager (Protein Simple), and plaque area was measured using ImageJ (80). Statistical significance was determined by Student’s *t* test (*P* < 0.05; GraphPad Prism); figures are representative of 2 independent experiments.

To determine bacterial doubling time, Henle-407 monolayers in 6-well plates were infected as described for plaque assays with S. flexneri grown in deoxycholate and added at a multiplicity of infection (MOI) of 20. At 1 h postinfection (hpi) and every 30 min thereafter, one well was trypsinized and Henle-407 cells were then lysed in 1% deoxycholate, diluted, and spot plated on TSBA-CR. Doubling time (*r*) was determined using the formula *t* × log_2_/(log *y*_2_ − log *y*_1_), where *t* is time (150 min), *y*_1_ is *Shigella* at 1 hpi, and *y*_2_ is *Shigella* at 3.5 hpi (*n* = 2).

Cell-to-cell spread assays were performed as previously described (81). S. flexneri was added to Henle-407 cells at approximately 65% confluence at an MOI of 10. Cells were stained with Wright-Giemsa stain at 3 hpi, and cell-to-cell spread rates were calculated by counting approximately 100 infected Henle-407 cells adjacent to other Henle-407 cells. The proportion of S. flexneri spreading events was scored by determining if any Henle-407 cells adjacent to an infected cell also contained 3 or more internal bacterial cells. Statistical significance was determined by Student’s *t* test (*n* = 3, *P* < 0.05).

Formate-induced cytotoxicity was determined by measuring secreted lactate dehydrogenase of Henle-407 cells supplemented with 20 mM formate every 24 h over 3 days using a lactate dehydrogenase assay (Sigma MAK066) according to the manufacturer’s instructions. Statistical significance was determined using ANOVA with a Bonferroni posttest.

### Formate uptake.

The formate uptake assay was modified from a previous study (22). Briefly, Henle-407 cells were cultured in 30- by 10-mm plates, and 20 μM [^14^C]formate (Moravek) was added to the tissue culture medium. Over the course of 48 h, cells were washed with PBS, trypsinized, and counted. Cells were then lysed directly in Optiphase HiSafe 3 (PerkinElmer). Counts per minute (cpm) was determined by scintillation, and counts were normalized to cell number and are relative to untreated cell lysates.

### Formate quantification.

For supernatant formate quantification, overnight cultures of S. flexneri WT and the indicated mutants were diluted 1:1,000 in M63 medium [100 mM KH_2_PO_4_, 15 mM (NH_4_)_2_SO_4_, 1.8 μM FeSO_4_, 1 mM MgSO_4_, 162.5 nM nicotinic acid, 10 mM glucose, 10 mM pyruvate]. Cultures were grown for approximately 18 h at 37°C in sealed anaerobe jars containing anaerobic gas packs (BD 260651). The OD_650_ was recorded to determine bacterial growth. Cultures were then centrifuged, supernatants were filter sterilized prior to analysis using a formate assay (Sigma MAK059) according to the manufacturer’s instructions, and samples were diluted 1:100 in formate assay buffer. Statistical significance was determined by ANOVA with a Bonferroni posttest (*P* < 0.05, *n* = 3) using GraphPad Prism.

For intracellular formate quantification, Henle-407 cells were infected with S. flexneri grown with deoxycholate as described above at an MOI of 5. At 5 hpi, Henle-407 monolayers were trypsinized and Henle-407 cells were counted using a Countess II cell counter (ThermoFisher). Where indicated, bacteria were quantified by plating. Cell pellets were then resuspended in 1 ml cold methanol-water (50:50) and incubated for 20 min on ice. Five hundred microliters of cold chloroform was added, and the mixture was vortexed and then centrifuged at 13,000 × *g* for 15 min. The aqueous phase was transferred to a new tube, liquid was evaporated using a vacuum manifold, and then samples were rehydrated in 100 μl formate assay buffer. Formate was quantified using the assay described above, and statistical significance was determined by either ANOVA with a Bonferroni posttest (*P* < 0.05, *n* = 3) or Student’s *t* test (*P* < 0.05, *n* = 3). Results are representative of 2 independent experiments.

### Phylogenetic analysis.

For the phylogenetic analysis of the PFL locus, the PFL locus was identified in the 79 *Shigella* complete genomes currently represented in the NCBI database. The sequences were aligned using Geneious and had 96.2% identical sites. The PFL loci from other related gastrointestinal pathogens were included for comparison. A tree was built using the Geneious Tree Builder, using the Tamura-Nei model and neighbor joining method, with global alignment with free end gaps and a cost matrix at 51% similarity. The tree was visualized using FigTree (http://tree.bio.ed.ac.uk/software/figtree).

### RNA extraction.

For *in vitro* RNA extractions, S. flexneri was grown in intracellular salts medium (ISM) (4) supplemented with 10 mM pyruvate, RDM supplement (82), 20 mM bicarbonate, and 100 μM nitrate. Five-milliliter cultures were grown statically at 37°C with 5% CO_2_ for 7 h. One milliliter of cold RNA-Stay (95% ethanol, 5% phenol) was added to each culture, and the entire volume was pelleted by centrifugation. One milliliter of cold RNA-Bee (Amsbio) was pipetted over the pellet. For RNA extractions of S. flexneri-infected Henle-407 cells, the Henle-407 cells were infected as described above with S. flexneri WT and mutants grown in deoxycholate and added at an MOI of 20. At 3 hpi, monolayers were washed once with PBS, and 1 ml of cold RNA-Bee (Amsbio) was pipetted over the monolayer. The cell lysates from either bacterial pellets or Henle-407 monolayers were transferred to new tubes, and 200 μl chloroform was added. The mixture was vortexed and after 5 min on ice was centrifuged at 13,000 × *g*, and the upper aqueous phase was transferred to a new tube. RNA was then precipitated with isopropanol, treated with DNase I (Invitrogen), and solubilized in water.

### cDNA library generation and RNA-seq.

RNA from infected Henle-407 cells grown with and without formate and a mock uninfected sample was used to generate cDNA by the Genomic Sequencing and Analysis Facility (GSAF) at the University of Texas at Austin. Single samples from each condition tested were analyzed. RNA was checked for quality using a Bioanalyzer (Agilent); samples had an RNA integrity number (RIN) of >9. One microgram of total RNA was used for ribosome depletion using the Ribo-Zero Gold rRNA removal kit (Illumina) according to the manufacturer’s instructions to eliminate both eukaryotic host rRNA and *Shigella* rRNA. The depleted RNA was fragmented to ∼200 bp, and cDNA was generated using the NEBNext Ultra II directional RNA kit. Samples were barcoded, and single-end sequencing (75 cycles) of cDNA libraries was performed using a NextSeq 500 system (Illumina) at the GSAF. Sequencing generated 1.1 × 10^8^ total reads for infected Henle-407 cells, and 1.0 × 10^8^ total reads for infected Henle-407 cells grown with formate.

### Read mapping and gene expression analysis.

Sequence reads were aligned to the S. flexneri 2457T genome (GenBank accession no. AE014073.1) and the S. flexneri virulence plasmid pCP301 (GenBank accession no. AF386526.1) using CLC Genomics Workbench (Qiagen). Multimapped reads were excluded, as were genes containing reads that mapped to the S. flexneri genome or virulence plasmid in the mock treatment (listed in Table S2). Values of reads per kilobase per million (RPKM) were used for expression analysis, and genes with an RPKM value of <20 (arbitrary cutoff) were excluded. A Baggerley proportions test with a false-discovery rate correction was used to determine statistical significance (*P* < 0.05). Genes with a fold change greater than 2 were considered significantly different. A complete list of read mappings can be found in Table S2.

10.1128/mBio.01777-18.9 TABLE S2 (A) Complete mapping information for S. flexneri genes induced by formate in the host cell listed in Table 1. (B) Mapping of all intracellular S. flexneri genes grown with and without formate. (C) Eighty-seven S. flexneri genes were mapped in mock-treated samples, to determine human RNA that maps to S. flexneri genes (false positives). Download Table S2, XLSX file, 1.5 MB. Copyright © 2018 Koestler et al. 2018 Koestler et al. This content is distributed under the terms of the Creative Commons Attribution 4.0 International license.

### Quantitative reverse transcriptase PCR.

RNA was quantified using a ND-1000 spectrophotometer (NanoDrop), and a total of 1 μg RNA was used with a high-capacity cDNA reverse transcription kit (Applied Biosystems). Two microliters of cDNA was then used as the template for SYBR green quantitative PCR (qPCR) (Applied Biosystems) using a ViiA 7 real-time PCR system (Applied Biosystems) at the University of Texas at Austin GSAF. Human gene expression was normalized to *actB*, and *Shigella* gene expression was normalized to *rssA*. All primers used for RT-qPCR are listed in Table S1. Statistical significance was determined using Student’s *t* test of threshold cycle (Δ*C_T_*) values compared to the S. flexneri WT-infected Henle-407 cells or S. flexneri WT grown in ISM.

### Fluorescence microscopy.

For IcsA labeling, Henle-407 cells were grown on glass coverslips in 6-well plates and infected with the S. flexneri WT, Δ*pflB*, or Δ*icsA* strain grown with deoxycholate (DOC) at an MOI of 1, as described above. At 3 hpi, cells were washed with PBS and then fixed with formaldehyde. Cells were then permeabilized with 0.5% Triton X-100, blocked with 5% bovine serum albumin (BSA) in PBS, and then incubated overnight with rabbit polyclonal antibody to IcsA (rabbit no. 5) provided by Edwin Oaks (Walter Reed Army Institute of Research) diluted 1:50 in PBS with 2.5% BSA. Cells were then washed with 0.05% Tween 20 in PBS and then blocked with 5% BSA in PBS for 30 min. The cells were then incubated with goat anti-rabbit IgG-FITC (sc-2012; Santa Cruz Biotechnology) diluted 1:1,000 and DAPI in PBS with 2.5% BSA for 2 h and then washed with 0.05% Tween 20 in PBS. Coverslips were then mounted on slides with Prolong Diamond antifade mountant (ThermoFisher). Images were then acquired using an Olympus BX41 microscope (100× objective) with a DP73 digital camera (Olympus) and processed using cellSens software (Olympus) or using a Zeiss LSM 710 confocal microscope (63× objective) at the University of Texas Microscopy and Imaging Facility. All exposures were identical, and images were processed in Photoshop (Adobe) to enhance contrast; all images were processed identically. Micrographs are representative of at least 3 independent experiments. To determine the proportion of intracellular bacteria with polar IcsA, the total number of bacteria and the number with polar IcsA staining were counted. Results shown are means ± standard deviations (SD) for the percent IcsA-positive bacteria in at least 4 fields.

S. flexneri strains containing the *ipaJ-gfp* reporter were grown with deoxycholate and ampicillin and diluted to an MOI of 1. Infections were carried out as described above, and at 3 hpi, cells were washed with PBS and formaldehyde fixed. Fixed cells were stained with wheat germ agglutinin (WGA)-Alexa Fluor 555 at 1 μg/ml. Slides were mounted with Vectashield with DAPI (Vector Laboratories), and images were acquired using an Olympus BX41 microscope (100× objective) with a DP73 digital camera (Olympus) and processed using cellSens software (Olympus). Acquisition of FITC (*gfp*) was fixed at 300 ms. Two-dimensional Henle-407 cell area was measured from the WGA membrane-labeled red channel, and *gfp* intensity of individual bacteria was quantified from the green channel using ImageJ (80). Background *gfp* fluorescence was measured in portions of each cell without bacteria and subtracted from individual bacterial cell fluorescence. A D’Agostino-Pearson normality test indicated that measurements did not conform to a Gaussian distribution. Therefore, statistical significance of *gfp* differences was determined by a Kruskal-Wallis test with a Dunn posttest (*P* < 0.05, *n* > 300). Regressions were performed using GraphPad Prism (*n* = 12 to 20), and slopes were found to be significantly different (*P* < 0.05).

10.1128/mBio.01777-18.7 FIG S7 Plaque size of S. flexneri WT, Δ*barA*, and Δ*uvrY* strains was measured; formate significantly increases WT plaque size 2.2-fold, increases Δ*barA* mutant plaque size 1.8-fold, and increases Δ*uvrY* mutant plaque size 2.0-fold. An asterisk indicates statistical significance. Download FIG S7, TIF file, 2.71 MB. Copyright © 2018 Koestler et al. 2018 Koestler et al. This content is distributed under the terms of the Creative Commons Attribution 4.0 International license.

### Accession number.

Sequencing data were deposited in GEO under accession number GSE119622.
