# Supplementary figures and images for: Formate Promotes Shigella Intercellular Spread and Virulence Gene Expression
Source: mBio. 2018 Sep 25;9(5):e01777-18. doi: 10.1128/mBio.01777-18 (PMC6156198; doi:10.1128/mBio.01777-18)

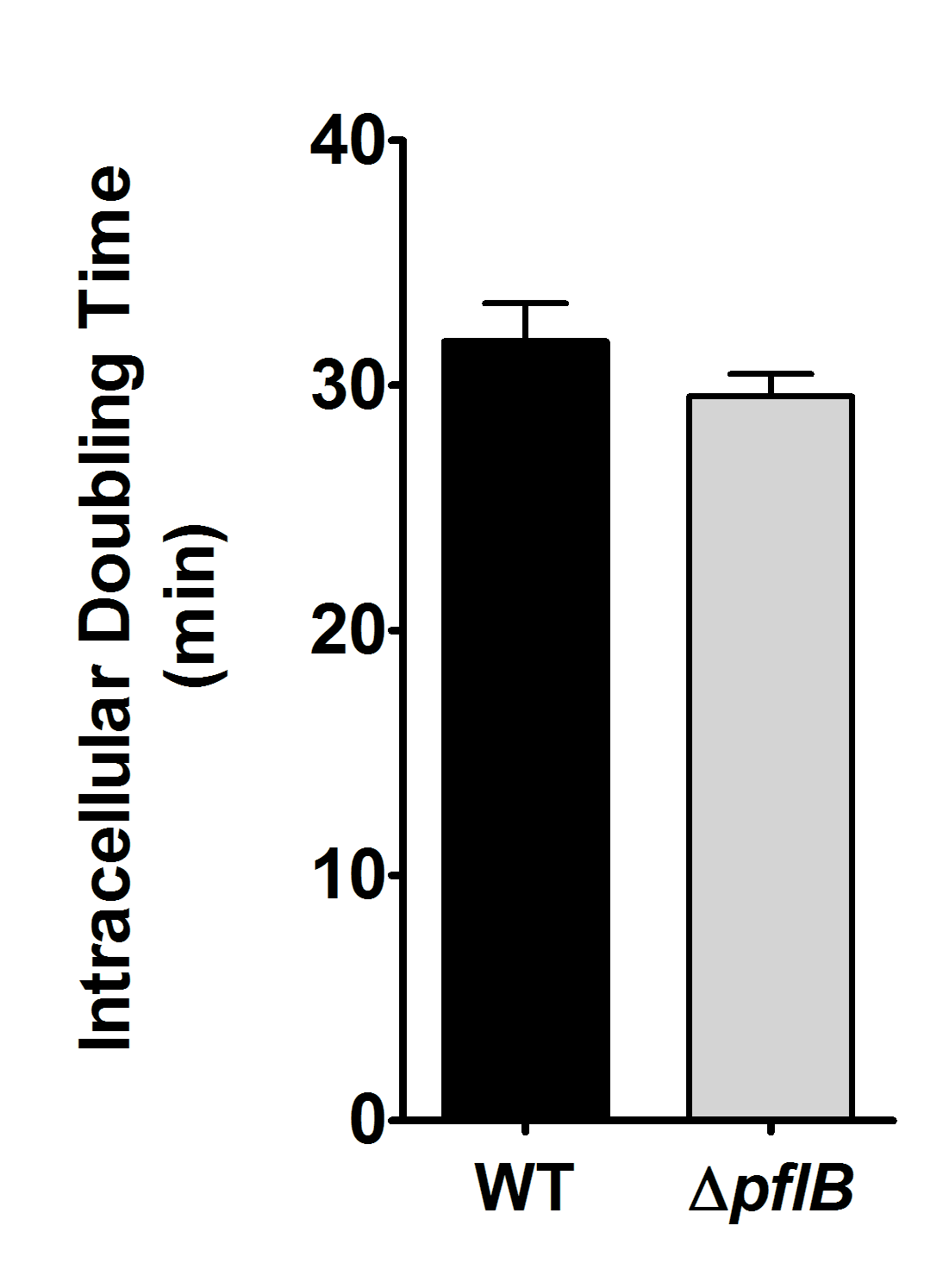

Supplement: FIG S1 [file mbo005184078sf1.tif]

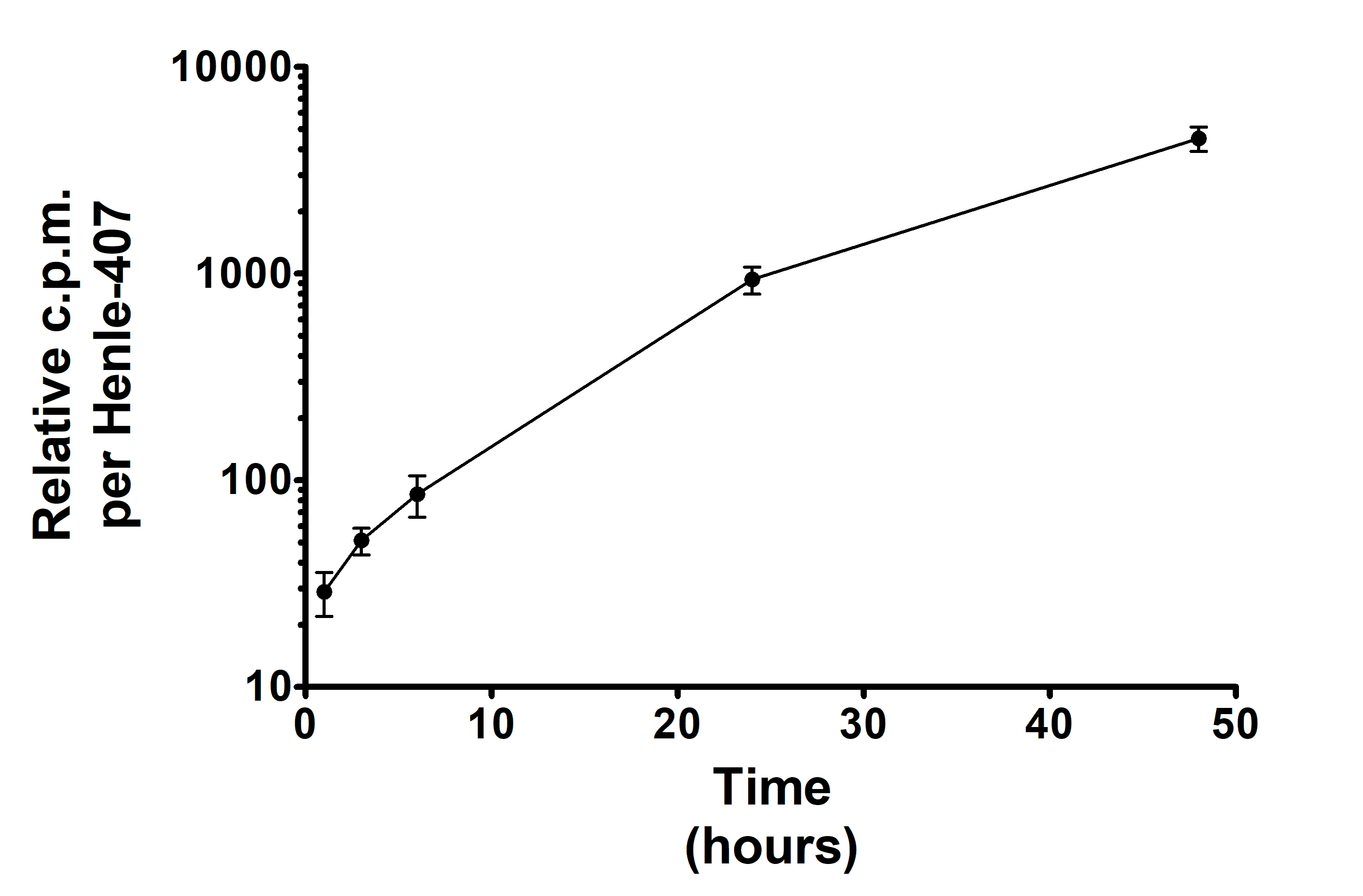

Supplement: FIG S2 [file mbo005184078sf2.tif]

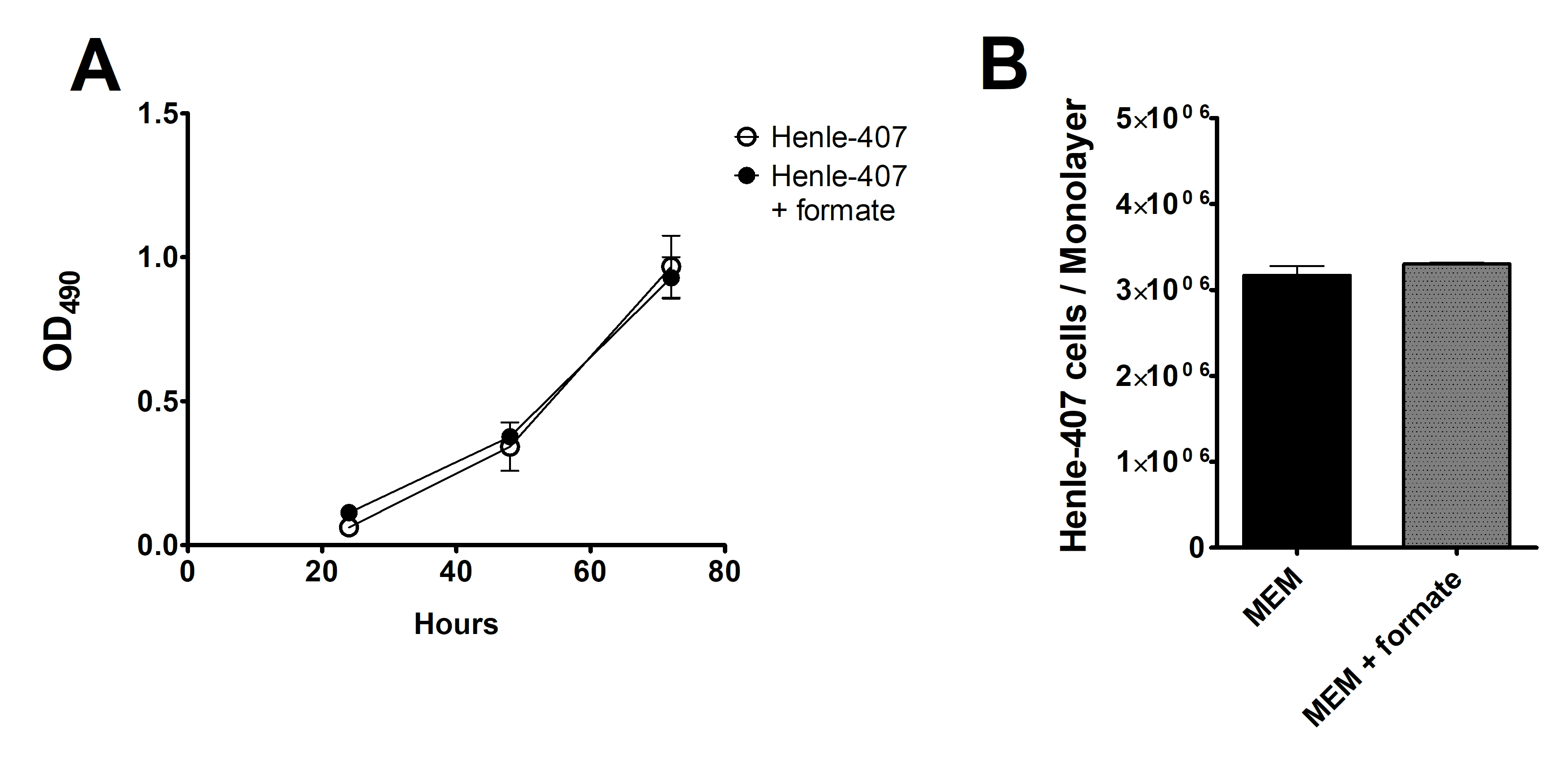

Supplement: FIG S3 [file mbo005184078sf3.tif]

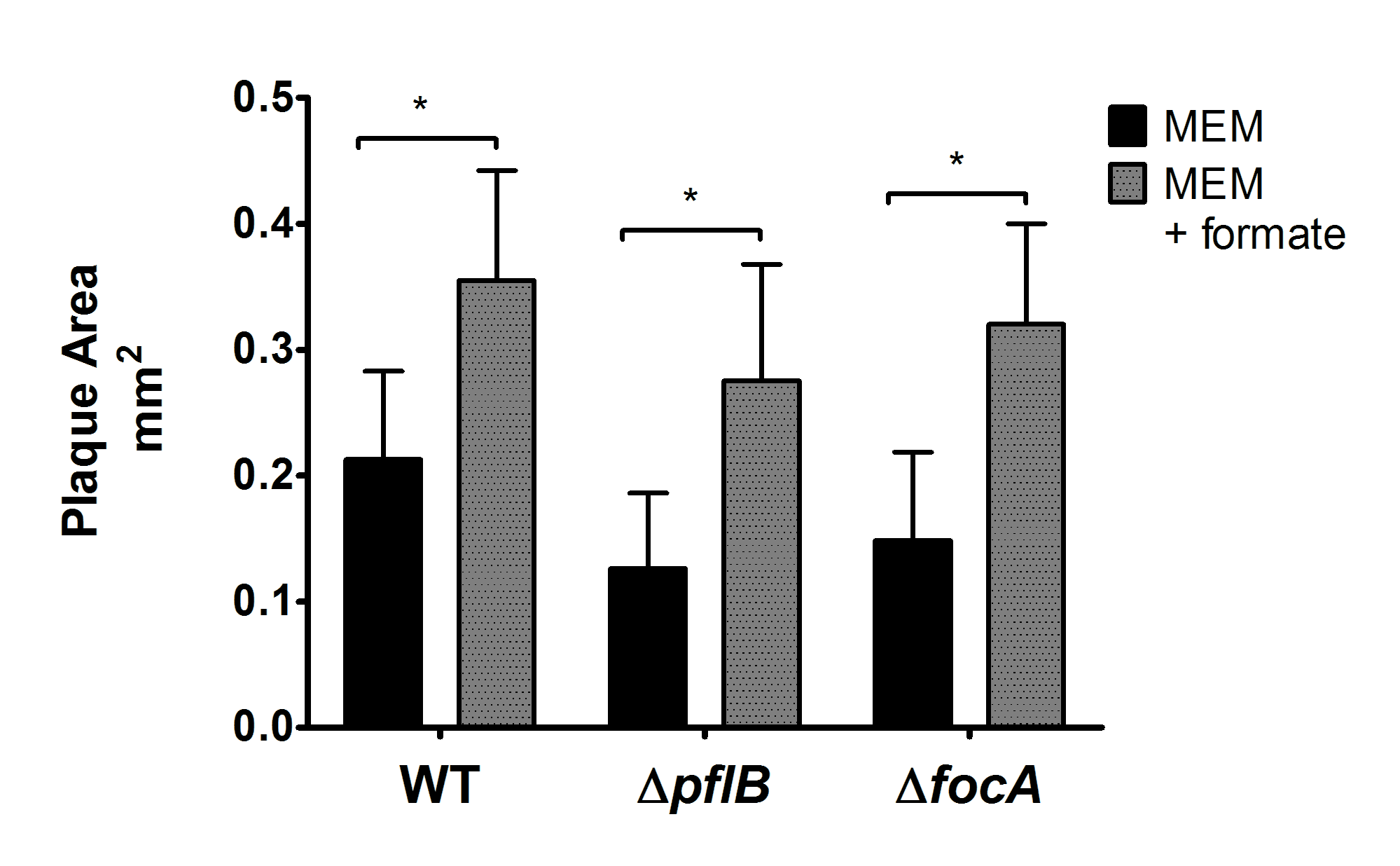

Supplement: FIG S4 [file mbo005184078sf4.tif]

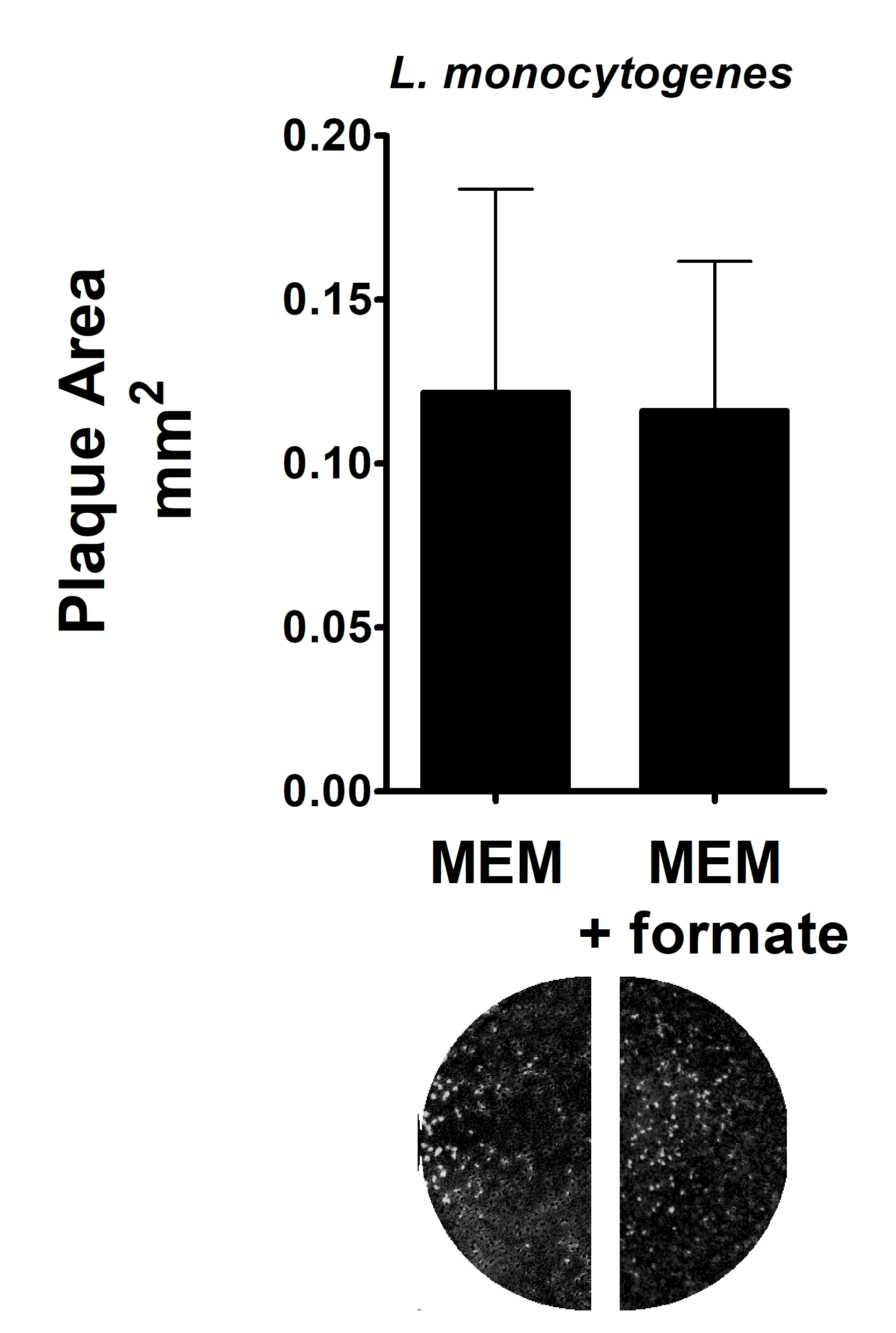

Supplement: FIG S5 [file mbo005184078sf5.tif]

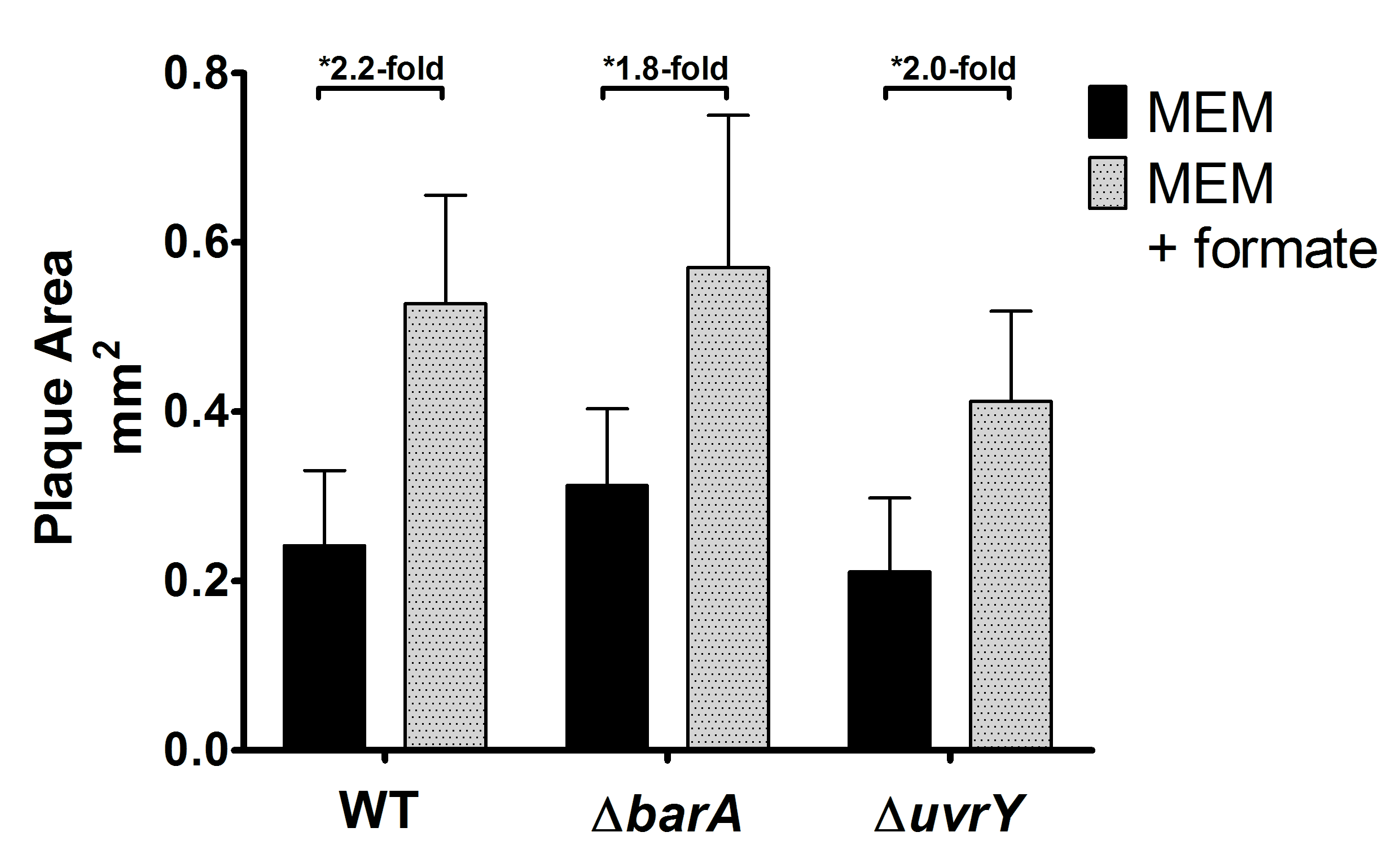

Supplement: FIG S7 [file mbo005184078sf7.tif]
