# Supplementary material for: Formate Promotes Shigella Intercellular Spread and Virulence Gene Expression
Source: mBio. 2018 Sep 25;9(5):e01777-18. doi: 10.1128/mBio.01777-18 (PMC6156198; doi:10.1128/mBio.01777-18)
Supplement: TABLE S1 [file mbo005184078st1.docx]

1. **Strains**

| Strain | Description | Source or reference |
| --- | --- | --- |
| *Human* |  |  |
| Henle-407 | HeLa contaminant cell line | ATCC CCL-6 (inestine 407) |
| CoN-841 | Untransformed colon epithelial cell line | ATCC CRL-1790 (CCD 841 CoN) |
|  |  |  |
| *Shigella flexneri* |  |  |
| 2457T | WT, Serotype 2a | Walter Reed Army Institute of Research |
| Δ*pflB* | 2457T Δ*pflB*::Kan | Pieper et al, 2013 (15) |
| Δ*fdnG* | 2457T Δ*fdnG*::Kan | This study |
| Δ*fdoG* | 2457T Δ*fdoG*::Kan | This study |
| ΔicsA | 2457T Δ*icsA*::E2-Tn5-Kan | Rossi et al, 2017 (46) |
| Δ*ipaJ* | 2457T Δ*ipaJ*::Cam | This study |
| Δ*ipgD* | 2457T Δ*ipgD*::Kan | Generous gift of C. Lesser |
| Δ*focA* | 2457T Δ*focA*::Kan | This study |
| Δ*barA* | 2457T ΔbarA::Kan | Gore and Payne, 2010 (64) |
| Δ*uvrY* | 2457T Δ*uvrY*::Kan | Gore and Payne, 2010 (64) |
|  |  |  |
| *Escherichia coli* |  |  |
| JW1470 | Keio Δ*fdnG*::Kan | Baba et al, 2006 (76) |
| JW3865 | Keio Δ*fdoG*::Kan | Baba et al, 2006 (76) |
| JW0887 | Keio Δ*focA*::Kan | Baba et al, 2006 (76) |
|  |  |  |
| *Listeria monocytogenes* | |  |
| *Listeria monocytogenes* EGDe | Serotype 1/2a | ATCC BAA-679 |

1. **Plasmids**

| Plasmid name | Lab designation | Plasmid backbone | Antibiotic resistance | Description | Source |
| --- | --- | --- | --- | --- | --- |
| pKD3 |  |  | cam | Template for camR cassette | Datsenko et al, 2000 (77) |
| pKD46 |  |  | amp | Plasmid with arabinose inducible recombinase | Datsenko et al, 2000 (77) |
| p-*ipaJ* | pBK24 | pWKS30 | amp | *ipaJ* complement plasmid containing *ipaJ* locus with native promoter | This study |
| p-*ipaJ*-*gfp* | pBK25 | pLR29 | amp | fusion of *ipaJ* promoter region to *gfp* | This study |
| p-ipgD | pACYC184+pipgD+ipgE | pACYC | cam | ipgD complement plasmid containing ipgD and ipgE with native promoter | Generous gift of M. Goldberg (49) |

1. **Primers**

| Primer name | Description | Sequence | | | |
| --- | --- | --- | --- | --- | --- |
| *Cloning* |  |  |  |  |  |
| fdnGseq-fw | sequencing fdnG knockout | 5' | CAGCCGATACAGGTGGAAACGTCGA | 3' |  |
| fdnGseq-rv | sequencing fdnG knockout | 5' | GCTTTTTGTCGTGTCATGCTCGCTG | 3' |  |
| fdoGseq-fw | sequencing fdoG knockout | 5' | CATCACGCCGCCATAACTCAATTTGACG | 3' |  |
| fdoGseq-rv | sequencing fdoG knockout | 5' | CCTGACAGGCTTTACAGCCGATACAGG | 3' |  |
| focAseq-fw | sequencing focA knockout | 5' | GCCAGTCACCTTTGGTAAAACCTTCCC | 3' |  |
| focAseq-rv | sequencing focA knockout | 5' | CCATGCGAGTTACGGGCCTATAAGC | 3' |  |
| ipaJKO-1 | Knocking out ipaJ | 5' | CCTGCGGATACTATCTATTCACTG | 3' |  |
| ipaJKO-2 | Knocking out ipaJ | 5' | GAAGCAGCTCCAGCCTACACCGACGAGTAATTGCAGCACAGTA | 3' |  |
| ipaJKO-3 | Knocking out ipaJ | 5' | TAAGGAGGATATTCATATGATTGTCGTTTTTTATCTGTGAAGGAGC | 3' |  |
| ipaJKO-4 | Knocking out ipaJ | 5' | CCAAAAGTTATGCTTGGTCAAAGAG | 3' |  |
| camR-fw | for amplifying camR from pKD3 (69) | 5' | GTGTAGGCTGGAGCTGCTTC | 3' |  |
| camR-rv | for amplifying camR from pKD3 (69) | 5' | CATATGAATATCCTCCTTA | 3' |  |
| ipaJseq-fw | sequencing ipaJ knockout | 5' | CGGCAGAGTTAATTGGAGAGCAC | 3' |  |
| ipaJseq-rv | sequencing ipaJ knockout | 5' | CTAATCTTCCCATAAAATGACCGACTTAC | 3' |  |
| ipaJ-30c-fw | cloning ipaJ complement into pWKS30, kpn1 site | 5' | ATAGGTACCCCTGCGGATACTATCTATTCACTGTGCTC | 3' |  |
| ipaJ-30c-rv | cloning ipaJ complement into pWKS30, xba1 site | 5' | ATATCTAGATTACAAAGCCTCATTAGTTATAACTATGG | 3' |  |
| pwKS30seq-1 | sequencing pWKS30 inserts | 5' | CCAGTGAGCGCGCGTAATACGAC | 3' |  |
| pwKS30seq-2 | sequencing pWKS30 inserts | 5' | CAGCTATGACCATGATTACGCC | 3' |  |
| ipaJ-gfp-fw | sma1 | 5' | ATACCCGGGGAATGAACTGGACATGTTGATAGACGG | 3' |  |
| ipaJ-gfp-rv | xba1 | 5' | ATATCTAGACGACGAGTAATTGCAGCACAGTATTG | 3' |  |
| pLR29seq-1 | sequencing for *gfp* reporter plasmid | 5' | CTGTTTCATATGATCTGGG | 3' |  |
| pLR29seq-2 | sequencing for *gfp* reporter plasmid | 5' | GAAGCAACGGCCCGGAG | 3' |  |
|  |  |  |  |  |  |
| *RT-qPCR* |  |  |  |  |  |
| actB-fw | Human actB qPCR primers | 5' | TCCCTGGAGAAGAGCTACG | 3' |  |
| actB-rv |  | 5' | GTAGTTTCGTGGATGCCACA | 3' |  |
| CXCL10-fw | Human CXCL10 qPCR primers | 5' | GAACTGTACGCTGTACCTGCA | 3' |  |
| CXCL10-rv |  | 5' | TTGATGGCCTTCGATTCTGGA | 3' |  |
| IL-8-fw | Human IL-8 qPCR primers | 5' | ATGACTTCCAAGCTGGCCGTGGCT | 3' |  |
| IL-8 rv |  | 5' | TCTAGCCCTCTTCAAAAACTTCTC | 3' |  |
| TNF-fw | Human TNF qPCR primers | 5' | GGAGAAGGGTGACCGACTCA | 3' |  |
| TNF-rv |  | 5' | CTGCCCAGACTCGGCAA | 3' |  |
| TNFAIP3-fw | Human TNFAIP3 qPCR primers | 5' | CCTTGGAAGCACCATGTTTG | 3' |  |
| TNFAIP3-rv |  | 5' | TTGTGTGGTTCGAGGCACAT | 3' |  |
| rrsA-fw | Shigella rrsA qPCR primers (67) | 5' | TGAAACGGATCCGCGCAAAGAAGTTGTC | 3' |  |
| rrsA-rv |  | 5' | ATGTTCTAGACCTGCCGCCAGCGTTC | 3' |  |
| ipaJ-fw | Shigella ipaJ qPCR primers | 5' | GCGAGGAAGCAGATGTATGAC | 3' |  |
| ipaJ-rv |  | 5' | CAACCCCTAATTCCTTTGCAG | 3' |  |
